# Intradialytic Exercise: One Size Doesn’t Fit All

**DOI:** 10.3389/fphys.2018.00844

**Published:** 2018-07-05

**Authors:** Pedro L. Valenzuela, Ana de Alba, Raquel Pedrero-Chamizo, Javier S. Morales, Fernando Cobo, Ana Botella, Marcela González-Gross, Margarita Pérez, Alejandro Lucia, M. T. Marín-López

**Affiliations:** ^1^Department of Systems Biology, Universidad de Alcalá, Madrid, Spain; ^2^Department of Sport and Health, Spanish Agency for Health Protection in Sport (AEPSAD), Madrid, Spain; ^3^Fundación Renal Íñigo Álvarez de Toledo, Madrid, Spain; ^4^Faculty of Physical Activity and Sport Sciences, Technical University of Madrid, Madrid, Spain; ^5^Faculty of Sport Sciences, European University of Madrid, Madrid, Spain; ^6^Research Institute i+12 and CIBER de Envejecimiento y Fragilidad (CIBERFES), Madrid, Spain

**Keywords:** hemodialysis, end-stage renal disease, chronic kidney disease, physical activity, training, mental health

## Abstract

**Purpose:** Hemodialysis patients commonly have impaired physical performance and mental health. We studied the effects of an intradialytic exercise program on these variables.

**Methods:** 27 patients (33% women; 68 ± 13 years) were enrolled in a 14-week intradialytic endurance-resistance training program (‘exercise’ group, 40 programmed sessions per patient); 40 hemodialysis patients (28% women; 68 ± 11 years) performing no exercise during the same time length were used as controls. Endpoints included physical performance (6-min walk test [6MWT], 10-repetition sit to stand [STS-10] and handgrip strength), emotional status (Beck’s depression inventory and State-Trait Anxiety Inventory), and mental and physical component scores of the short-from (SF)-12 Health Survey.

**Results:** There were no differences (*p*> 0.05) between groups at baseline for sex distribution, or mean age, body mass index and time spent on dialysis. Exercise benefits were observed for 6MWT (11 and −3% for the exercise and control groups, respectively; *p* < 0.001), STS-10 performance time (-22 and 6%; *p* < 0.001) and handgrip strength (4 and −4%; *p* < 0.02). No significant benefits (*p*> 0.05) were observed for emotional status endpoints or SF-12 component scores. Despite significant benefits on physical performance, the proportion of clinically meaningful responders was low (<50%). Responsiveness was dependent on baseline physical performance (*p* < 0.05) but not on age or sex (*p* > 0.05).

**Conclusion:** A 14-week intradialytic training program induced significant improvements on physical performance. However, the rate of clinically meaningful responders observed in the present study was low, being the level of responsiveness dependent on baseline physical status. Efforts to individualize exercise prescription are needed in clinical practice.

## Introduction

The prevalence of patients with end-stage renal disease is rapidly growing, especially among the elderly population and patients with comorbidities (particularly diabetes mellitus and hypertension) (Szczech and Lazar, 2004). Consequently, more than two million people are expected to be treated by dialysis for end-stage renal disease by 2030 (Szczech and Lazar, 2004).

Despite important progress in hemodialysis techniques and in the treatment of its associated comorbidities, patients have a much higher morbimortality risk than their healthy counterparts (Foley et al., 1998; Szczech and Lazar, 2004). Dialysis is associated with a deterioration of physical function and mental status. Muscle function (Leal et al., 2011b) and exercise capacity (Painter, 2005) are significantly lower in hemodialysis patients, presenting a peak oxygen consumption that is considerably lower (>50%) compared to their healthy sedentary peers (Painter, 2005). Emotional disorders such as anxiety and depression are prevalent among dialysis patients (Dziubek et al., 2016; King-Wing Ma and Kam-Tao Li, 2016), negatively affecting their social, financial and psychological well-being, as well as their quality of life (QoL) (Christensen and Ehlers, 2002).

Physical fitness is one of the strongest predictors of survival in dialysis patients, with low levels of physical activity and impaired physical performance being associated with increased mortality risk in this population (O’Hare et al., 2003; Sietsema et al., 2004; Stack et al., 2005; Roshanravan et al., 2013; Torino et al., 2014; Morishita et al., 2017). In addition, lower QoL and mental health are also strongly associated with higher risk of death and hospitalization (Knight et al., 2003; Mapes et al., 2003). Therefore, maintaining their physical and mental status closer to their healthy counterparts is of major importance.

Meta-analytical evidence supports the benefits of intradialytic exercise programs for the improvement of several health-related outcomes such as physical performance or mental health (Smart and Steele, 2011; Chung et al., 2017). Yet, exercise benefits in dialytic patients are typically reported under the assumption that the group average represents the response of most individuals. However, a wide interindividual variability can be observed in the human response to a similar training program, which results in subjects being classified as responders (those who achieve clinically meaningful benefits) or non-responders (those who experience a worsening or remain unchanged) (Mann et al., 2014). The aim of this study was to analyze the effects of a 14-week intradialytic combined exercise (endurance + resistance) training program on patients’ mental and health status. In addition, we assessed the influence of baseline phenotype on the training response as well as individual variability in training responses to the study endpoints.

## Materials and Methods

### Participants and Study Design

End-stage-renal disease patients undergoing hemodialysis were recruited for the study. Subjects were excluded if they presented one or more of the following conditions: myocardial infarction in the 6 weeks prior to the start of the exercise program, unstable angina, cerebrovascular disease or a high risk for recurrence, musculoskeletal or respiratory (e.g., chronic obstructive pulmonary disease) alterations, uncontrolled hypertension, peripheral vascular disease, active liver disease, osteoporosis, cardiac ejection fraction <45%, blood hemoglobin concentration <10 g/dL, or problematic vascular access (immature arteriovenous fistulas, high risk for extravasation). All participants had the procedures explained and provided written informed consent to participate in the study. The present study was approved by the institutional review board (P141115303, *Fundación Universitaria Hospital de Alcorcón*, Madrid, Spain).

The study took place between January 2015 and May 2016. Patients in the ‘exercise’ group had to participate in a 14-week intradialytic training program, whereas those of the ‘control’ group had to maintain their regular lifestyle during this time period without direct intervention from the personnel of this investigation. From a total of 235 patients undergoing hemodialysis in different dialysis centers, two cohorts of 86 and 74 patients met the inclusion criteria to participate as ‘exercise’ and ‘control’ group, respectively. From these, 12 and 31 patients, respectively, did not participate. Reasons not to participate were receiving a transplant, leaving the center, not signing the informed consent form after having the study explained to them, and not being interested. For the rest of patients, only those who performed at least two physical tests and two psychological tests at baseline were enrolled in the study. Finally, 27 and 40 patients were included in the exercise and control group, respectively. Participants’ descriptive data are presented in **Table 1**.

**Table 1:** Descriptive baseline characteristics of the participants.

	Control	Exercise	*p*-value
Women (%)	28	33	0.79
Age (years)	68 ± 11	68 ± 13	0.92
BMI (kg⋅m^−2^)	27 ± 5	27 ± 6	0.99
Dialysis prescription (hours⋅week^−1^)	11 ± 1	11 ± 1	0.51
Time on dialysis (years)	5 ± 4	7 ± 5	0.08

*Data are Mean ± SD. Abbreviations: BMI, body mass index.*

### Exercise Intervention

The intradialytic training intervention consisted of 14 weeks of combined endurance and resistance exercises. Training sessions were conducted at three different dialysis centers but were supervised by the same experienced fitness instructors. Training sessions were performed three times per week and lasted approximately 60 min. A total of 40 training sessions were planned per subject during the intervention portion of the study.

Training sessions started with a warm-up consisting of respiratory and joint mobility exercises. During the main part of the sessions, both resistance and endurance exercises were performed. Resistance exercises included ankle plantarflexion and dorsiflexion, combined knee and hip flexion and extension, hip abduction and adduction, and abdominal exercises. These exercises were performed using elastic bands, Styrofoam balls and ankle weights. Endurance exercise consisted of pedaling on a mini bike for 30 min at an intensity corresponding to 12–14 points in the Borg’s 6–20 scale (Borg, 1998).

### Endpoints

Endpoints were assessed the week before (baseline) and after (post-intervention) the 14-week intervention. Assessment was done on dialysis days, with each participant being tested at the same time of the day (i.e., always in the morning or in the afternoon, before starting dialysis). Before the testing sessions, participants were individually instructed on how to perform all tests with detailed explanations and visual examples. Two testing sessions per patient were required to perform all the tests at each time point, one for all physical performance tests and another one for psychological evaluation. The tests were always performed in the same order.

### Physical Performance

We assessed patients’ performance in the 10-repetition sit to stand (STS-10), handgrip strength and 6-min walk (6MWT) tests (performed in this order), which are some of the most popular fitness tests in dialysis patients (Koufaki and Kouidi, 2010) and present an excellent test-retest reliability in this population (Segura-Ortí and Martínez-Olmos, 2011).

The STS-10, an index of lower-extremity strength (Csuka and McCarty, 1985), measures the time (in seconds) required to perform 10 consecutive repetitions of sitting down and getting up from a chair. Participants began the test with their arms crossed on their chest and sitting with their back against the chair. They were instructed to perform the task “as fast as possible,” starting and finishing at the sitting position. Time was measured with a stopwatch (ONstart 100, Geonaute, France) to the nearest 0.1 s. This test has previously demonstrated a good test-retest reliability in hemodialysis patients (intra-class correlation coefficient [ICC] = 0.88) (Segura-Ortí and Martínez-Olmos, 2011).

Maximal isometric handgrip force has been suggested as a useful tool for the continuous assessment of muscle mass and function in dialysis patients (Leal et al., 2011a). It was measured in both hands using a manual dynamometer (T.K.K.5401, Takei Scientific Instruments, Japan) while participants were in a standing position, with the arm extended and parallel to the body, and without moving the wrist. They performed two maximal repetitions with each hand interspersed with 1-min rest periods between trials, and the mean of all four trials (combined handgrip strength) was analyzed. This test has also proven highly reliable in hemodialysis patients (ICC = 0.95 and 0.96 for the dominant and non-dominant hand, respectively) (Segura-Ortí and Martínez-Olmos, 2011).

The 6MWT was used as a marker of endurance capacity (Rikli and Jones, 1998). It was performed on a 17-meter corridor with marks on every meter, and time was measured with a chronometer (ONstart 100, Geonaute, France). Participants were asked to cover the greatest distance possible during 6 min by walking (not running) continuously and turning around at the final mark. No verbal encouragement was given during the test; however, feedback regarding the remaining time was available. Participants were allowed to rest during the test, and to use any ambulation aid (e.g., crutches) that they used during daily life. A very high test-retest reliability has been previously reported for this test in hemodialysis patients (ICC = 0.94) (Segura-Ortí and Martínez-Olmos, 2011).

### Mental and Health Status

Changes in depression symptoms were assessed using the Beck Depression Inventory (BDI) (Beck et al., 1996). In this self-reported questionnaire 21 items are rated on a four-point severity scale and summed to give a total score, with a higher score being suggestive of more severe depression. The BDI has proven a valid depression screening tool in dialysis patients (Preljevic et al., 2012), being one of the most commonly used questionnaires to assess this condition in this patient population (King-Wing Ma and Kam-Tao Li, 2016). This questionnaire has previously yielded high values of internal consistency (Cronbach’s α = 0.89), sensitivity (0.82) and specificity (0.87–0.89) in dialysis patients (Preljevic et al., 2012). Test–retest coefficients in other populations have been reported to range from 0.62 (7-week interval) to 0.93 (1-week interval) (Julian, 2011).

Health-related QoL (HRQoL) was assessed using the Short-Form 12 (SF-12) health survey, a short version of the SF-36 (Ware and Sherbourne, 1992). A physical (PCS) and a mental component score (MCS) are calculated from this self-reported questionnaire. SF-12 has previously proven reliable in a 6-month longitudinal study performed with dialysis patients (ICC = 0.90 and 0.86 for MCS and PCS, respectively) (Loosman et al., 2015). Moreover, SF-12 scores are associated with short-term and long-term mortality in this population (Loosman et al., 2015).

The level of anxiety was assessed using the State-Trait Anxiety Inventory (STAI) (Spielberger et al., 1970). We specifically analyzed the anxiety subscale. This test has previously shown a good internal consistency (Cronbach’s α = 0.86–0.95) and reliability over time (*r* = 0.65–0.75) (Spielberger et al., 1970; Julian, 2011).

### Statistical Analysis

All the participants assessed at baseline were considered to be part of the study. Missing individual data at post-intervention were imputed with the ‘baseline-observation-carried forward’ method, that is, baseline values were used when these data were missing. The normal distribution (Shapiro-Wilk test) and homoscedasticity (Levene’s test) of the data were checked before any statistical treatment. Non-normally distributed data (results from STAI and BDI) were log-transformed prior to its analysis. Differences in proportions were evaluated using Pearson’s chi-squared test. Differences in baseline characteristics were analyzed using unpaired Student’s *t*-tests. Endpoints were analyzed by a two-way mixed ANOVA with time points (baseline and post-intervention) as the within-subject factor and intervention groups (control or exercise) as the between-subject factor. The effect size (partial eta-squared, ${\text{η}}_{\text{p}}^{2}$) of the significant group x time interactions was calculated and considered small (>0.01) moderate (>0.06) or large (>0.14) (Cohen, 1988). *Post hoc* analysis (Bonferroni test) was conducted when a significant interaction (group × time) effect was found.

The rate of clinically meaningful responders was calculated in those endpoints in which a beneficial effect of exercise (i.e., significant group x time interaction) was found. Responsiveness was defined as beneficial changes that exceeded two times the standard error of measurement (SEM) (Hopkins, 2000). The responsiveness threshold for the physical tests was set at 3 kg, 7.2 s, and 56.8 m for handgrip strength, STS-10 and 6MWT, respectively, attending to the SEM values previously reported for these tests in dialysis patients (Segura-Ortí and Martínez-Olmos, 2011). The magnitude of the differences (effect size, ES) in baseline values between responders and non-responders was determined through standardized mean differences (Hedges’ g). Pearson’s correlation analyses (for physical performance and age) and Pearson’s chi-square test (for sex) were used to determine the influence of baseline phenotype on training responsiveness. All analyses were performed using a statistical Package (SPSS, version 23.0).

## Results

There were no significant differences between control and exercise groups in baseline characteristics (**Table 1**). All subjects in the exercise group completed at least 80% of the planned training sessions. No major adverse events or health-related issues attributable to exercise were noted.

Four subjects in each group could not complete the baseline 10-STS assessment due to excessive weakness or mobility limitations (i.e., use of crutches), and therefore the sample analyzed for this test was of 36 and 23 for the control and the exercise group, respectively. After the 14-week intervention four subjects in the control group could not perform the 10-STS and one subject in this same group could not perform the psychological tests, and thus we used their baseline values.

No significant changes in physical performance measures were observed in the control group between baseline and post-intervention. By contrast, a significant improvement was observed in the exercise group for 6MWT (*p* = 0.006, ES = 0.31), STS-10 (*p* < 0.001, ES = 0.59) and combined handgrip strength (*p* = 0.027, ES = 0.12). Significant interactions (group × time) with moderate to large effect sizes were found for all physical performance measures (**Table 2**). *Post hoc* analyses revealed significant differences between groups at post-intervention for 6MWT (*p* = 0.005), STS-10 (*p* < 0.001) and combined handgrip strength (*p* = 0.017).

**Table 2:** Effects of an intradialytic exercise program on markers of physical and mental health.

End point	Group	n with baseline data	Baseline	Post-intervention	Change (95% CI)	Group × Time effect	Effect size (${\text{η}}_{\text{p}}^{2}$)^a^
**6MWT (m)**	Control	40	341 ± 127	330 ± 118	−11 (−27, 5)	**0.001**	0.160
	Exercise	27	380 ± 131	422 ± 136	42 (13, 70)		
**STS-10 (s)**	Control	36	32 ± 11	34 ± 12	2 (−1, 5)	**<0.001**	0.203
	Exercise	23	26 ± 10	21 ± 8	−6 (−8, −4)		
**Handgrip (kg)**	Control	40	25 ± 8	24 ± 8	−1 (−2, 0)	**0.02**	0.084
	Exercise	27	28 ± 8	29 ± 8	1 (0, 2)		
**STAI-S**	Control	40	18 ± 13	18 ± 12	0 (−2, 2)	0.10	–
	Exercise	27	19 ± 9	17 ± 10	−2 (−5, 2)		
**BDI**	Control	40	15 ± 13	14 ± 10	−1 (−3, 2)	0.32	–
	Exercise	27	10 ± 8	8 ± 7	−2 (−4, −1)		
**PCS**	Control	40	61 ± 17	66 ± 16	5 (0, 10)	0.36	−
	Exercise	27	62 ± 20	63 ± 23	1 (−5, 8)		
**MCS**	Control	40	70 ± 20	73 ± 16	3 (−1, 8)	0.54	–
	Exercise	27	75 ± 14	76 ± 15	1 (−3, 5)		

*Data are mean ± SD. Significant p-values are highlighted in bold. Handgrip strength corresponds to the mean of the two arms. Abbreviations: 6MWT, six minute-walk test; 95% CI, 95% confidence intervals; BDI, Beck’s depression inventory; MCS, mental component summary; PCS, physical component summary; STAI-S, State-Trait Anxiety Inventory; STS-10, sit to stand test. 4 and 1 subjects of the control group could not perform the STS-10 and the psychological tests, respectively, at post-intervention, and their baseline values were used. Data from STAI-S and BDI were log-transformed prior to its analysis, but are shown as raw data for the sake of clarity.*

**FIGURE 1:**
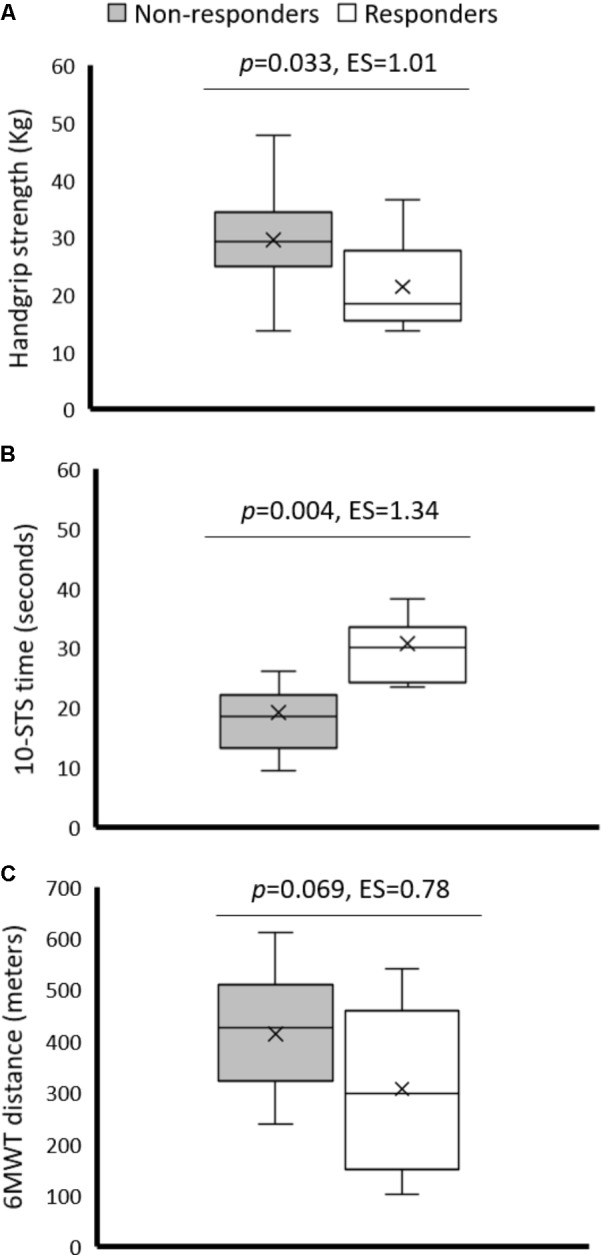
Differences in baseline performance for combined handgrip strength **(A)** 10-repetition sit to stand **(B)** and 6-min walk test **(C)** between responders and non-responders to the intradialytic training program.

**FIGURE 2:**
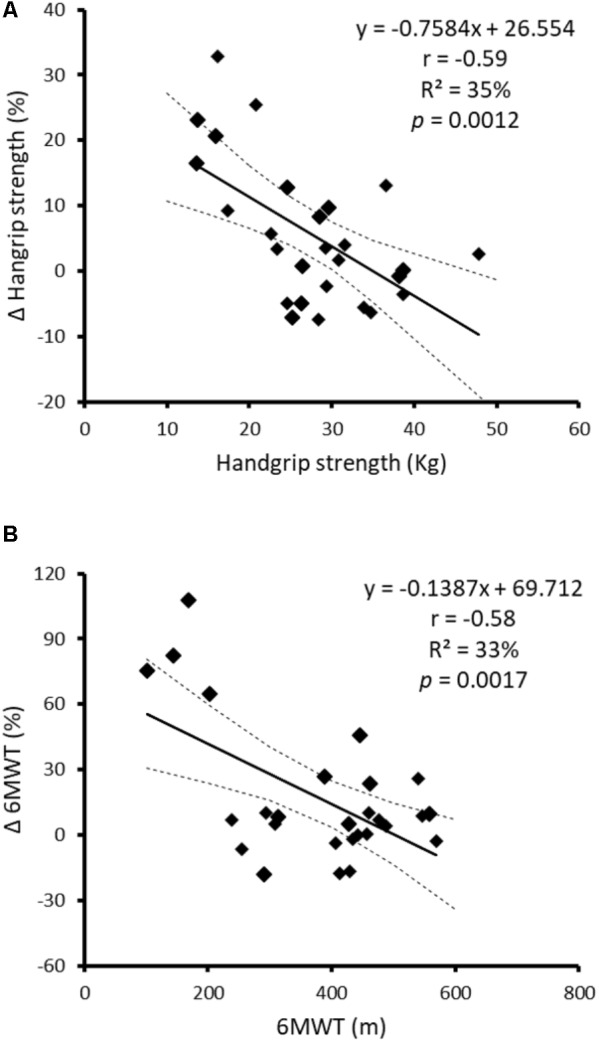
Relationship between baseline performance for combined handgrip strength **(A)** and 6-min walk distance **(B)** with the change observed in these tests after the intradialytic training program. Solid and dashed lines represent regression line and 90% confidence intervals, respectively.

Despite statistically significant benefits, only 30, 46, and 20% of subjects in the exercise group were clinically meaningful responders for 6MWT, STS-10 and handgrip strength test, respectively. Of note, responsiveness was dependent on baseline physical fitness, that is, participants with lower baseline physical fitness showed greater improvements. Indeed, significant differences were found between responders and non-responders for baseline physical performance (**Figure 1**), and a significant inverse relationship was observed between baseline combined handgrip strength and 6MWT, on one hand, and the relative performance improvement in these tests, on the other (**Figure 2**). There were no significant differences between sexes for the rate of responders observed in 6MWT (33 and 22% for men and women, respectively; *p* = 0.882), STS-10 (53 and 33%, respectively; *p* = 0.597) or handgrip strength test (11 and 44%, respectively; *p* = 0.141). No significant relationship (*p*> 0.05) was observed between age and relative improvement on physical performance for any test.

Regarding mental status, BDI scores significantly decreased in the exercise group at post-intervention compared to baseline (*p* = 0.006, ES = 0.29), whereas no significant changes were observed in the control group (**Table 2**). No significant differences (*p*> 0.05) between baseline and post-intervention were observed for any of the other mental and health status endpoints in the control or exercise group (**Table 2**). No significant interaction (group x time) was found for any of the mental and health status endpoints (**Table 2**).

## Discussion

The present results show that a 14-week intradialytic training program including endurance and resistance exercise induced improvements in mean values of physical performance, which are significantly lower in this population than in their healthy counterparts (Painter, 2005; Leal et al., 2011b). Specifically, significant improvements were observed for the average value of 6MWT, a valid predictor of mortality, cardiovascular events and hospitalization in dialysis patients (Torino et al., 2014). Exercise training also resulted in an increased strength of the lower limb muscles (as reflected by a lower time on average to complete the STS-10 test), which is important because an impaired physical performance of the lower extremities is strongly associated with all-cause mortality in these patients (Roshanravan et al., 2013). We also found an exercise-training induced improvement in handgrip strength, with decreases in this variable being related to a decreased inflammatory status and higher muscle mass and survival expectancy in this population (Leal et al., 2011a). Therefore, these results are of major clinical importance, as they suggest that an intradialytic exercise program can attenuate dialysis-associated physical impairment and thus might also potentially reduce morbimortality risk in these patients (Morishita et al., 2017).

The effectiveness of intradialytic training programs for the improvement of physical performance has been previously demonstrated (Smart and Steele, 2011; Chung et al., 2017). Although the response to exercise interventions is commonly described in general terms under the assumption that the group average represents the response of most individuals (Mann et al., 2014), it has now been demonstrated that a considerable individual variability can be observed even in tightly controlled studies (Yan et al., 2017). In this context, an interesting finding of the present study is that, despite significant improvements in mean physical performance, the rate of clinically meaningful responders was overall low (<50%). Several hypotheses have been proposed to account for individual variability in response to exercise training (Mann et al., 2014). In our study, baseline physical performance − but not participants’ age or sex − partly conditioned the level of responsiveness to the training program, with the less fit patients at baseline being those showing greater benefits. These results suggest that the training stimulus was high enough to induce clinically meaningful improvements in less fit subjects but not in their fitter peers. Notwithstanding, even non-responders presented a lower physical performance at baseline than expected for their age (Casanova et al., 2011; Massy-Westropp et al., 2011). Therefore, efforts to enhance responsiveness in these subjects are needed, which might probably involve applying a higher training stimulus (e.g., higher intensity or volume) (Mann et al., 2014).

Although in agreement with our results some studies have found no changes in variables such as HRQoL or depression after intradialytic training programs (van Vilsteren et al., 2005; Parsons et al., 2006), most studies have reported benefits on these psychological variables (Suh et al., 2002; Levendoğlu et al., 2004; Ouzouni et al., 2009; Dziubek et al., 2016; Frih et al., 2017). Interestingly, the level of anxiety observed in our patients was overall low, with only 4% of subjects in the intervention group presenting a STAI score higher than 40, which is the proposed cut-off for detecting clinically significant symptoms of anxiety) (Knight et al., 1983; Julian, 2011). HRQoL was also surprisingly good, with the observed mean MCS and PCS being higher than those previously reported in other dialysis populations (Mapes et al., 2003; Lacson et al., 2010; Frih et al., 2017). The lack of significant differences in these variables in our study might have been due to the low prevalence of psychological disorders in the analyzed sample, which can be a result of the psychological therapy that all subjects received since they started dialysis. Nevertheless, a significant reduction of 23% in mean BDI scores was observed after the exercise program in the present study, and a reduction of >17.5% has proven to be the threshold above which depressive individuals report feeling better (Button et al., 2015). Therefore, the observed benefits of exercise on depression levels could be of clinical importance despite no statistically significant differences between groups.

Considering the importance of physical activity and performance for dialysis patients (O’Hare et al., 2003; Sietsema et al., 2004; Stack et al., 2005; Matsuzawa et al., 2012; Roshanravan et al., 2013) and their low levels of physical activity (Johansen et al., 2010), promoting physical activity in this population should be a priority. Intradialytic exercise programs have proven safe and effective not only for improving physical performance (Smart and Steele, 2011; Chung et al., 2017) but also dialysis efficacy (Parsons et al., 2006), and therefore these programs should be routinely included in clinical practice. Nevertheless, the present study highlights the need of individualizing training programs so as to achieve an optimal stimulus for every patient.

Our study has some limitations, including mainly the lack of subjects’ familiarization sessions with the tests and the fact that we did not perform a randomized controlled trial. In addition, several potential confounders which were not considered here have been proposed to influence inter-individual variability in response to a training stimulus. Particularly a commonly overlooked source of error is within-subject variability, with recent research providing some insights into its importance (Hecksteden et al., 2015, 2018; Lindholm et al., 2016) and another ongoing project, the Gene Smart study, currently embracing this concept (Yan et al., 2017). However, applying the designs that allow to control for confounders like within-subject variability (e.g., performing repeated tests both before and after the intervention, or using a crossover study with repeated training intervention) might not be feasible in patient populations such as the present one. While keeping the aforementioned limitations in mind, a major strength and novelty of our approach was the individualized analysis of training responses, which allowed us to estimate the rate of clinically meaningful responders.

## Conclusion

A 14-week intradialytic endurance-resistance training program improved patients’ physical performance on average. Yet, baseline physical status affected the level of responsiveness to the training program, with only those patients presenting the lowest physical fitness at the beginning of the intervention obtaining clinically meaningful benefits from the training program. Efforts to individualize exercise prescription are needed in clinical practice to enhance responsiveness. Future reach might determine if applying a higher training stimulus (i.e., higher intensity or volume) in the fitter subjects actually results in a clinically meaningful response.

## Ethics Statement

This study was carried out in accordance with the recommendations of the institutional review board of Fundación Universitaria Hospital de Alcorcón (Madrid, Spain). The protocol was approved by the institutional review board of Fundación Universitaria Hospital de Alcorcón (Madrid, Spain). All subjects gave written informed consent in accordance with the Declaration of Helsinki.

## Author Contributions

AB, RP-C, MG-G, and MP conceived, designed, and supervised the study. AdA, FC, and MM-L supervised the training sessions and performed the evaluations. PV and JM analyzed the data. PV drafted the manuscript. All authors significantly contributed to the final version of the manuscript.

## Conflict of Interest Statement

The authors declare that the research was conducted in the absence of any commercial or financial relationships that could be construed as a potential conflict of interest.

## References

[B1] Beck A. Steer R. A. Brown G. K. (1996). *Beck Depression Inventory*, 2nd Edn. San Antonio, TX: The Psycological Corporation.

[B2] Borg G. (1998). *Borg’s Perceived Exertion and Pain Scales*, 7th Edn. Champaign, IL: Human Kinetics.

[B3] Button K. S. Kounali D. Thomas L. Wiles N. J. Peters T. J. Welton N. J. (2015). Minimal clinically important difference on the Beck Depression Inventory-II according to the patient’s perspective. *Psychol. Med.* 45 3269–3279. 10.1017/S0033291715001270 26165748 PMC4611356

[B4] Casanova C. Celli B. R. Barria P. Casas A. Cote C. De Torres J. P. (2011). The 6-min walk distance in healthy subjects: reference standards from seven countries. *Eur. Respir. J.* 37 150–156. 10.1183/09031936.00194909 20525717

[B5] Christensen A. J. Ehlers S. L. (2002). Psychological factors in end-stage renal disease: an emerging context for behavioral medicine research. *J. Consult. Clin. Psychol.* 70 712–724. 10.1037//0022-006X.70.3.712 12090378

[B6] Chung Y. C. Yeh M. L. Liu Y. M. (2017). Effects of intradialytic exercise on the physical function, depression and quality of life for haemodialysis patients: a systematic review and meta-analysis of randomised controlled trials. *J. Clin. Nurs.* 26 1801–1813. 10.1111/jocn.13514 27532211

[B7] Cohen J. (1988). *Statistical Power Analysis for the Behavioral Sciences*, 2nd Edn. Hillsdale, NJ: Lawrence Erlbaum Associates. 10.1234/12345678

[B8] Csuka M. McCarty D. J. (1985). Simple method for measurement of lower extremity muscle strength. *Am. J. Med.* 78 77–81. 10.1016/0002-9343(85)90465-6 3966492

[B9] Dziubek W. Kowalska J. Kusztal M. Rogowski Ł. Gołêbiowski T. Nikifur M. (2016). The level of anxiety and depression in dialysis patients undertaking regular physical exercise training - a preliminary study. *Kidney Blood Press. Res.* 41 86–98. 10.1159/000368548 26872253

[B10] Foley R. Parfrey P. Sarnak M. (1998). Clinical epidemiology of cardiovascular disease in chronic renal disease. *Am. J. Kidney Dis.* 32 S112–S119. 10.1053/ajkd.1998.v32.pm9820470 9820470

[B11] Frih B. Jaafar H. Mkacher W. Ben Salah Z. Hammami M. Frih A. (2017). The effect of interdialytic combined resistance and aerobic exercise training on health related outcomes in chronic hemodialysis patients: the Tunisian randomized controlled study. *Front. Physiol.* 8:288. 10.3389/fphys.2017.00288 28620308 PMC5449721

[B12] Hecksteden A. Kraushaar J. Scharhag-Rosenberger F. Theisen D. Senn S. Meyer T. (2015). Individual response to exercise training – a statistical perspective. *J. Appl. Physiol.* 118 1450–1459. 10.1152/japplphysiol.00714.2014 25663672

[B13] Hecksteden A. Pitsch W. Rosenberger F. Meyer T. (2018). Repeated testing for the assessment of individual response to exercise training. *J. Appl. Physiol.* 10.1152/japplphysiol.00896.2017 [Epub ahead of print]. 29357481

[B14] Hopkins W. G. (2000). Measures of reliability in sports medicine and science. *Sports Med.* 30 1–15. 10.2165/00007256-200030010-00001 10907753

[B15] Johansen K. L. Chertow G. M. Kutner N. G. Dalrymple L. S. Grimes B. A. Kaysen G. A. (2010). Low level of self-reported physical activity in ambulatory patients new to dialysis. *Kidney Int.* 78 1164–1170. 10.1038/ki.2010.312 20811334 PMC4170106

[B16] Julian L. J. (2011). Measures of Anxiety. *Arthritis Care* 63 0–11. 10.1002/acr.20561 22588767 PMC3879951

[B17] King-Wing Ma T. Kam-Tao Li P. (2016). Depression in dialysis patients. *Nephrology (Carlton)* 21 639–646. 10.1111/nep.12742 26860073

[B18] Knight E. L. Ofsthun N. Teng M. Lazarus J. M. Curhan G. C. (2003). The association between mental health, physical function, and hemodialysis mortality. *Kidney Int.* 63 1843–1851. 10.1046/j.1523-1755.2003.00931.x 12675862

[B19] Knight R. G. Waal Manning H. J. Spears G. F. (1983). Some norms and reliability data for the State–Trait Anxiety Inventory and the Zung Self-Rating Depression scale. *Br. J. Clin. Psychol.* 22 245–249. 10.1111/j.2044-8260.1983.tb00610.x 6640176

[B20] Koufaki P. Kouidi E. (2010). Current best evidence recommendations on measurement and interpretation of physical function in patients with chronic kidney disease. *Sports Med.* 40 1055–1074. 10.2165/11536880-000000000-00000 21058751

[B21] Lacson E. Xu J. Lin S. F. Dean S. G. Lazarus J. M. Hakim R. M. (2010). A comparison of SF-36 and SF-12 composite scores and subsequent hospitalization and mortality risks in long-term dialysis patients. *Clin. J. Am. Soc. Nephrol.* 5 252–260. 10.2215/CJN.07231009 20019120 PMC2827595

[B22] Leal V. O. Mafra D. Fouque D. Anjos L. A. (2011a). Use of handgrip strength in the assessment of the muscle function of chronic kidney disease patients on dialysis: a systematic review. *Nephrol. Dial. Transplant.* 26 1354–1360. 10.1093/ndt/gfq487 20709742

[B23] Leal V. O. Stockler-Pinto M. B. Farage N. E. Aranha L. N. Fouque D. Anjos L. A. (2011b). Handgrip strength and its dialysis determinants in hemodialysis patients. *Nutrition* 27 1125–1129. 10.1016/j.nut.2010.12.012 21454052

[B24] Levendoğlu F. Altintepe L. L. Okudan N. Uğurlu H. Gökbel H. Tonbul Z. (2004). A twelve week exercise program improves the psychological status, quality of life and work capacity in hemodialysis patients. *J. Nephrol.* 17 826–832. 15593058

[B25] Lindholm M. E. Giacomello S. Werne Solnestam B. Fischer H. Huss M. Kjellqvist S. (2016). The impact of endurance training on human skeletal muscle memory, global isoform expression and novel transcripts. *PLoS Genet.* 12:e1006294. 10.1371/journal.pgen.1006294 27657503 PMC5033478

[B26] Loosman W. L. Hoekstra T. Van Dijk S. Terwee C. B. Honig A. Siegert C. E. H. (2015). Short-Form 12 or Short-Form 36 to measure quality-of-life changes in dialysis patients? *Nephrol. Dial. Transplant.* 30 1170–1176. 10.1093/ndt/gfv066 25829325

[B27] Mann T. N. Lamberts R. P. Lambert M. I. (2014). High responders and low responders: factors associated with individual variation in response to standardized training. *Sports Med.* 44 1113–1124. 10.1007/s40279-014-0197-3 24807838

[B28] Mapes D. L. Lopes A. A. Satayathum S. McCullough K. P. Goodkin D. A. Locatelli F. (2003). Health-related quality of life as a predictor of mortality and hospitalization: the Dialysis Outcomes and Practice Patterns Study (DOPPS). *Kidney Int.* 64 339–349. 10.1046/j.1523-1755.2003.00072.x 12787427

[B29] Massy-Westropp N. M. Gill T. K. Taylor A. W. Bohannon R. W. Hill C. L. (2011). Hand Grip Strength: age and gender stratified normative data in a population-based study. *BMC Res. Notes* 4:127. 10.1186/1756-0500-4-127 21492469 PMC3101655

[B30] Matsuzawa R. Matsunaga A. Wang G. Kutsuna T. Ishii A. Abe Y. (2012). Habitual physical activity measured by accelerometer and survival in maintenance hemodialysis patients. *Clin. J. Am. Soc. Nephrol.* 7 2010–2016. 10.2215/CJN.03660412 22977216 PMC3513746

[B31] Morishita S. Tsubaki A. Shirai N. (2017). Physical function was related to mortality in patients with chronic kidney disease and dialysis. *Hemodial. Int.* 21 483–489. 10.1111/hdi.12564 28418625

[B32] O’Hare A. M. Tawney K. Bacchetti P. Johansen K. L. (2003). Decreased survival among sedentary patients undergoing dialysis: results from the dialysis morbidity and mortality study wave 2. *Am. J. Kidney Dis.* 41 447–454. 10.1053/ajkd.2003.50055 12552509

[B33] Ouzouni S. Kouidi E. Sioulis A. Grekas D. Deligiannis A. (2009). Effects of intradialytic exercise training on health-related quality of life indices in haemodialysis patients. *Clin. Rehabil.* 23 53–60. 10.1177/0269215508096760 19114437

[B34] Painter P. (2005). Physical functioning in end-stage renal disease patients: update 2005. *Hemodial. Int.* 9 218–235. 10.1111/j.1492-7535.2005.01136.x 16191072

[B35] Parsons T. L. Toffelmire E. B. King-VanVlack C. E. (2006). Exercise training during hemodialysis improves dialysis efficacy and physical performance. *Arch. Phys. Med. Rehabil.* 87 680–687. 10.1016/j.apmr.2005.12.044 16635631

[B36] Preljevic V. T. Østhus T. B. H. Sandvik L. Opjordsmoen S. Nordhus I. H. Os I. (2012). Screening for anxiety and depression in dialysis patients: comparison of the Hospital Anxiety and Depression Scale and the Beck Depression Inventory. *J. Psychosom. Res.* 73 139–144. 10.1016/j.jpsychores.2012.04.015 22789418

[B37] Rikli R. E. Jones C. J. (1998). The reliability and validity of a 6-minute walk test as a measure of physical endurance in older adults. *J. Aging Phys. Act.* 6 363–375. 10.1097/00005768-199805001-199805421

[B38] Roshanravan B. Robinson-Cohen C. Patel K. V. Ayers E. Littman A. J. de Boer I. H. (2013). Association between physical performance and all-cause mortality in CKD. *J. Am. Soc. Nephrol.* 24 822–830. 10.1681/ASN.2012070702 23599380 PMC3636794

[B39] Segura-Ortí E. Martínez-Olmos F. J. (2011). Test-retest reliability and minimal detectable change scores for sit-to-stand-to-sit tests, the six-minute walk test, the one-leg heel-rise test, and handgrip strength in people undergoing hemodialysis. *Phys. Ther.* 91 1244–1252. 10.2522/ptj.20100141 21719637

[B40] Sietsema K. E. Amato A. Adler S. G. Brass E. P. (2004). Exercise capacity as a predictor of survival among ambulatory patients with end-stage renal disease. *Kidney Int.* 65 719–724. 10.1111/j.1523-1755.2004.00411.x 14717947

[B41] Smart N. Steele M. (2011). Exercise training in haemodialysis patients: a systematic review and meta-analysis. *Nephrology (Carlton)* 16 626–632. 10.1111/j.1440-1797.2011.01471.x 21557787

[B42] Spielberger C. D. Gorsuch R. Lushene R. (1970). *Test Manual for the State-Trait Anxiety Inventory.* Palo Alto, CA: Consulting Psychologists Press.

[B43] Stack A. G. Molony D. A. Rives T. Tyson J. Murthy B. V. R. (2005). Association of physical activity with mortality in the US dialysis population. *Am. J. Kidney Dis.* 45 690–701. 10.1053/j.ajkd.2004.12.013 15806472

[B44] Suh M. R. Jung H. Kim S. Park J. Yang W. (2002). Effects of regular exercise on anxiety, depression, and quality of life in maintenance hemodialysis patients. *Ren. Fail.* 24 337–345. 10.1081/JDI-120005367 12166700

[B45] Szczech L. A. Lazar I. L. (2004). Projecting the United States ESRD population: issues regarding treatment of patients with ESRD. *Kidney Int.* 66 (Suppl. 90), S3–S7. 10.1111/j.1523-1755.2004.09002.x 15296500

[B46] Torino C. Manfredini F. Bolignano D. Aucella F. Baggetta R. Barillà A. (2014). Physical performance and clinical outcomes in dialysis patients: a secondary analysis of the EXCITE trial. *Kidney Blood Press. Res.* 39 205–211. 10.1159/000355798 25118076

[B47] van Vilsteren M. C. B. A. de Greef M. H. G. Huisman R. M. (2005). The effects of a low-to-moderate intensity pre-conditioning exercise programme linked with exercise counselling for sedentary haemodialysis patients in The Netherlands: results of a randomized clinical trial. *Nephrol. Dial. Transplant.* 20 141–146. 10.1093/ndt/gfh560 15522901

[B48] Ware J. E. Sherbourne C. D. (1992). The MOS 36-item short-form health survey (SF-36). Conceptual framework and item selection. *Med. Care* 30 473–483. 10.1097/00005650-199206000-00002 1593914

[B49] Yan X. Eynon N. Papadimitriou I. D. Kuang J. Munson F. Tirosh O.‘ (2017). The gene SMART study: method, study design, and preliminary findings. *BMC Genomics* 18:821. 10.1186/s12864-017-4186-4184 29143594 PMC5688409

